# Circadian gene hClock enhances proliferation and inhibits apoptosis of human colorectal carcinoma cells *in vitro* and *in vivo*

**DOI:** 10.3892/mmr.2015.3247

**Published:** 2015-01-26

**Authors:** YAPING WANG, RUIZHE QIAN, NING SUN, CHAO LU, ZONGYOU CHEN, LUCHUN HUA

**Affiliations:** 1 Department of Surgery, Huashan Hospital, Fudan University, Shanghai 200040, P.R. China; 2 Department of Physiology and Pathophysiology, Shanghai Medical College, Fudan University, Shanghai 200032, P.R. China

**Keywords:** circadian, human circadian locomotor output cycles kaput gene, colorectal carcinoma, apoptosis

## Abstract

Colorectal carcinoma (CRC) is one of the most prevalent types of malignancy-associated mortality worldwide. Previous studies have demonstrated that amplification and overexpression of the human circadian locomotor output cycles kaput gene (hClock) was closely associated with a high risk for CRC as well as poor prognosis in CRC patients. However, the underlying molecular mechanisms of CRC remain to be fully elucidated. In the present study, hClock was exogenously overexpressed in the CRC cell line SW480 via infection of a lentivirus vector expressing hClock; in addition, a lentivirus vector-based RNA interference approach, using short hairpin RNA, was performed in order to knockdown hClock in SW620 cells. The results showed that upregulation of hClock promoted proliferation and inhibited apoptosis in SW480 cells *in vitro* and *in vivo*, while downregulation of hClock inhibited SW620 cell proliferation and accelerated apoptosis *in vitro*. Upregulation of hClock enhanced the activity of the anti-apoptotic gene phosphorpylated (p-) AKT and inhibited the expression of the pro-apoptotic gene B cell lymphoma-2 (Bcl-2)-associated X protein and Bcl-2 homology 3 interacting domain death agonist. Furthermore, targeted inhibition of hClock activity reduced p-AKT expression. In conclusion, the results of the present study suggested that the circadian gene hClock promoted CRC progression and inhibit tumor cell apoptosis *in vitro* and *in vivo*, while silencing hClock was able to reverse this effect.

## Introduction

Daily rhythms, which are essential for normal behavior and numerous physiological processes, including the sleep-wake cycle, hormone secretion, core body temperature, metabolism and cell cycle control are generated by the mammalian circadian timing system (1). These circadian clock rhythms are controlled at the molecular level through interactions between positive and negative feedback loops, which consist of various important clock regulators (2,3). One molecular clock model, which has been well established, encompasses a feedback system that involves the heterodimer transcriptional factors Clock and Bmal1, two cryptochromes (Cry1 and Cry2) and three period regulator genes (Per1, Per2 and Per3); among these, Clock acts as the master controller gene and serves as the positive regulator in the primary feedback loop (4).

Previous studies have provided evidence which suggested that disruption of the circadian rhythm or genetic defects in the circadian core genes were associated with cancer biology (5,6). In addition, disturbances of circadian gene expression levels have been reported in various types of human malignancies (7–13). Soták *et al* (14) demonstrated that circadian genes may have a role in colorectal carcinoma (CRC) development and progression. The circadian rhythmicity of certain circadian genes, including Per1, Per2 and Bmal1, was significantly reduced in CRC tumor tissue compared with that of a healthy colon (14). CRC is the most common type of solid tumor worldwide (15); however, further studies are required in order to fully elucidate the molecular mechanisms of CRC and to control this cancer more effectively (16,17).

It was previously reported that the circadian gene human Clock (hClock) was highly expressed in CRC tissues compared with that of peritumoral tissues in CRC patients, and was found to be strongly associated with late tumor-node-metastasis (TNM) stage as well as positive lymph node metastasis (18). Similar studies have also demonstrated the overexpression of the hClock gene in human CRC tissues (19,20). Furthermore, Alhopuro *et al* (21) reported that ~53% of microsatellite instability CRCs had point mutations in the hClock gene DNA sequence and Zhou *et al* (22) showed that genetic variants in the hClock gene have a significant effect on the risk of mortality in CRC patients. Overall, these results strongly suggested a critical role for hClock in CRC progression. However, the function of hClock in CRC remains to be fully elucidated. It has been estimated that up to 10% of all genes within the mammalian genome are regulated by circadian genes (23). Numerous genes regulating the cell cycle, including Wee1, cyclin D1 and c-Myc, have been reported to be controlled by the rhythmic activity of core clock genes (24–26). Molecular clockworks regulate the expression of these clock-controlled genes associated with the cell cycle, apoptosis and other pathways in cells; therefore, the aberration of core circadian genes, such as hClock, may result in the dysregulation of these processes and lead to tumor development (27,28).

The aim of the present study was to investigate the molecular changes and function of hClock in human CRC progression through hClock upregulation and knock-down experiments *in vitro* and *in vivo*.

## Materials and methods

### Ethics statement

The present study was approved by the Institutional Review Board of Huashan Hospital affiliated to Fudan University (HIRB; Shanghai, China). All *in vivo* experiments were performed strictly in accordance with the National Institutes of Health Guide for the Care and Use of Laboratory Animals and were approved by Animal Care and Use Committee of Shanghai Medical College of Fudan University (Shanghai, China).

### Cell culture

The two human CRC cell lines SW480 and SW620, as well as the human embryonic kidney cell line 293T were cultured in Dulbecco’s modified Eagle’s medium (DMEM; Gibco Life Technologies, Carlsbad, CA, USA) with 10% (v/v) newborn calf serum (Amresco LLC, Solon, OH, USA), 100 U/ml penicillin and 100 ng/ml streptomycin (both Amresco LLC) at 37°C in 5% CO_2_. All cell lines were obtained from the Cell Bank of the Chinese Academy of Sciences (Shanghai, China).

### Western blot analysis

Cells were homogenized in ice-cold radio immunoprecipitation assay lysis buffer (Beijing Dingguo Biotechnology Co., Ltd., Beijing, China). Following centrifugation (12,000 × g, 10 min at 4°C), the supernatant was collected and the protein concentration was determined using a bicinchoninic acid protein assay kit (HyClone-Pierce, Logan, UT, USA). Equal amounts of proteins (40 *μ*g) were extracted and separated using 10% SDS-PAGE gels, the proteins were then electrotransferred to a polyvinylidene fluoride membrane (Pall-Gelman, Port Washington, NY, USA). The membrane was blocked with 5% fat-free milk powder for 1 h, and then incubated with the primary antibodies (1:500 dilution) and GAPDH monoclonal antibody (1:1,000 dilution) in blocking buffer for 18 h at 4°C. The membranes were subsequently incubated with a secondary antibody (1:4,000 dilution) for 1 h at room temperature. After washing, immunodetection was performed with enhanced chemiluminescence (ECL-PLUS/kit; Amersham, Piscataway, NJ, USA) and exposed on an X-ray film. Subsequently, the image was processed and analyzed by ImageJ (v2.1.4.7). GAPDH was used as the loading control. The antibodies anti-hClock (cat. no. ab43106), rabbit monoclonal anti-B cell lymphoma (Bcl-2)-associated X protein (Bax; cat. no. ab32503) and mouse monoclonal anti-Bcl-2 homology 3 interacting domain death agonist (Bid; cat. no. ab114051) were obtained from Abcam (Cambridge, UK), while rabbit monoclonal anti-GAPDH (cat. no. 3683), anti-AKT (cat. no. 4685) and rabbit monoclonal anti-phosphorylated (p-)AKT (cat. no. 3783) were obtained from Cell Signaling Technology, Inc. (Danvers, MA, USA). The secondary antibody was alkaline phosphatase-labeled anti-rabbit immunoglobulin G (Beijing Dingguo Biotechnology Co., Ltd.).

### Plasmid construction

The full-length complementary (c)DNA of hClock (Genbank accession number, NM_004898) was amplified and inserted into the pcDNA3-Flag vector (Invitrogen Life Technologies, Carlsbad, CA, USA) in order to generate the pcDNA3-Flag-hClock expression plasmid. The cDNA fragment of Flag-hClock was then inserted into pGV186 retroviral vector (Shanghai GeneChem Co., Ltd., Shanghai, China) to generate pGV186-Flag-hClock. All the constructs were confirmed by DNA sequencing, which was performed by Shanghai GeneChem Co., Ltd.

### RNA interference

For small interfering (si)RNA-mediated hClock silencing, the following target siRNA sequences of hClock (NM_004898) were used: hClock RNAi-1, 5′-ACGAGAACTTGGCATTGAA-3′; hClock RNAi-2, 5′-CAAGATTCTGGGTCAGATA-3′; hClock RNAi-3, 5′-CACACATAGGCCATCTTAT-3′; and hClock RNAi-4, 5′-TTCAACTTTCTTCTGGAAA-3′.

The RNA duplexes were synthesized by Genepharma Co., Ltd (Shanghai, China). Then, a pGV113 lentiviral vector, which had an independent open reading frame of red fluorescence protein, was used to produce double-stranded siRNA. In order to construct the hairpin siRNA expression cassette, cDNA nucleotides of hClock siRNA were synthesized, annealed and inserted into pGV113 by Shanghai GeneChem Co., Ltd. A total of four groups of pGV113-hClock small hairpin (sh)RNA were constructed (Shanghai GeneChem Co., Ltd, Shanghai, China). The targeting sequences for hClock were as follows: hClock RNAi-1, 5′-CCGGCGACGAGA ACTTGGCATTGAACTCGAGTTCAATGCCAAGTTCTCGTCGTTTTTG-3′; hClock RNAi-2, 5′-CCGGGCCAAGATTCTGGGTCAGATACTCGAGTATCTGACCCAGAATCTTGGCTTTTTG-3′; hClock RNAi-3, 5′-CCGGCGCACACATAGGCCATCTTATCTCGAGATAAGATGGCCTATGTGTGCGTTTTTG-3′; and hClock RNAi-4, 5′-CCGGCGTTCAACTTTCTTCTGGAAACTCGAGTTTCCAGAAGAAAGTTGAACGTTTTTG-3′. Lentiviral vector pGV113 (Shanghai GeneChem Co., Ltd.) was used as a negative control.

### Retrovirus packing, transduction and infection of target cells

Retroviruses carrying the hClock cDNA or hClock siRNA were generated by co-transfection of recombinant pGV186 or pGV113 plasmids, respectively, with pHelper1.0 and pHelper2.0 plasmids (Shanghai GeneChem Co., Ltd.) into 293T cells using Lipofectamine 2000 (Invitrogen Life Technologies). These 293T cells were then cultured in DMEM supplemented with 10% newborn calf serum in a 37°C incubator containing 5% CO_2_. Forty-eight hours post-transfection, the supernatant was collected by centrifugation at 1,000 × g for 10 min, and the culture medium containing the recombinant virus was harvested and purified using a a 0.45-*μ*m filter (Shanghai GeneChem Co., Ltd.). Target cells were seeded (5×10^5^/well) into six-well plates and incubated with recombinant virus supplemented with 5 *μ*g/ml polybrene (Shanghai GeneChem Co., Ltd.) for a spin infection procedure. The SW480 cells were transduced with the retroviruses containing hClock or control sequence plasmids, while the SW620 cells were transduced with the retroviruses containing hClock siRNA or control sequence plasmids. Two weeks after infection, the stable cells infected with the target retrovirus were selected.

### Cell viability assay

Following 12 h of transfection, the MTT standard method was used to determine the cell growth. Cells were seeded into the 96-well plates at a density of 1,000 cells/well and cultured at 37°C in a humidified atmosphere containing 5% CO_2_. Following 96 h, the cells were incubated with 100 *μ*g/well MTT solution (Sigma-Aldrich, St. Louis, MO, USA) for 4 h, the medium was replaced with 150 *μ*l dimethyl sulfoxide (DMSO; Sigma-Aldrich) and agitated for 10 min. Absorbance was recorded at 490 nm using an automatic microwell plate reader (3350; Bio-Rad Laboratories, Inc., Hercules, CA, USA). Cell viability was calculated as percentage of that of the untreated cells.

### Flow cytometric analysis

Cells were harvested through 0.25% trypsin (Amresco LLC) digestion at 72 h post-seeding, and then washed with ice-cold phosphate-buffered saline (Sigma-Aldrich). Cells were re-suspended in 100 *μ*l 1X binding buffer (Invitrogen Life Technologies) at a concentration of 1×10^6^ cells/ml and double stained with Annexin V and propidium iodide (PI; Invitrogen Life Technologies). Following 30 min of incubation at room temperature in the dark, cells were added to 400 *μ*l 1X binding buffer and immediately analyzed by flow cytometry (FACS Calibur; BD Biosciences, San Jose, CA, USA).

### Tumor growth in xenograft models

A total of 20 female Balb/c nude mice (4–5 weeks old, ~20 g) were obtained from Shanghai Experimental Animal Center and maintained in a pathogen-free environment. The mice were divided at random into two groups (n=10/group), housed two per cage and provided with food and water *ad libitum*. All of the mice were exposed to a 12 h light/12 h dark cycle. The two groups of mice were subcutaneously injected with SW480-hClock or SW480-control infected cells (1×10^6^) in each right flank. The tumors which developed were measured weekly using a caliper, and the diameters were recorded. Tumor volume (cm^3^) was calculated using the following formula: Length × width^2^ × 0.5326. The animals were sacrificed by cervical dislocation six weeks post-injection of cells. The tumors were then resected and snap-frozen in liquid nitrogen for further analysis. Tumor tissues were fixed in 10% formalin for at least 24 h, then embedded in paraffin wax and sectioned (4 *μ*m) for histopathological evaluation. The sections were stained with hematoxylin and eosin using a standard protocol and analyzed by light microscopy (DM IL LED; Leica, Wetzlar, Germany). All animal procedures in the present study were conducted in accordance with the guidelines of the Institutional Animal Care and Use Committee. All mice were treated humanely throughout the experimental period.

### Statistical analysis

Values are presented as mean ± standard deviation, unless stated otherwise. Statistical significance of the *in vitro* and *in vivo* studies was analyzed using Student’s *t*-test. All statistical analyses were conducted using SPSS 19.0 software (International Business Machines, Armonk, NY, USA). All P-values were two-sided and P<0.05 was considered to indicate a statistically significant difference between values.

## Results

### hClock promotes CRC cell proliferation in vitro

**Figure 1:**
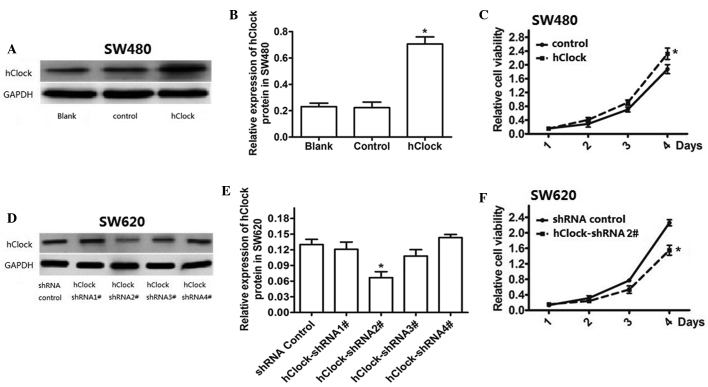
Effects of hClock on CRC cell proliferation. (A) Western blot analysis was performed to confirm the successful stable overexpression of the hClock gene in SW480 cells following transfection with pGV186-hClock-lentivirus and (B) hClock protein expression was normalized to that of GAPDH. Blank, untreated SW480 cells; control, pGV186-lentivirus transduced SW480 cells. (C) MTT assay examining proliferation of SW480 CRC cells following overexpression of hClock. (D) Western blot analysis of hClock protein expression in hClock shRNA-transfected (hClock shRNA1#-4#) and vector only-transfected SW620 cells. (E) hClock protein expression levels were normalized to GAPDH expression. hClock shRNA Clone 2# showed significant downregulation of hClock protein expression compared with that of control vector-transduced cells and was therefore selected for further analysis. (F) MTT assay showed that silencing of hClock expression inhibited proliferation of SW620 CRC cells. Values are presented as the mean ± standard deviation (n=3). ^*^P<0.05 vs. control or shRNA control. hClock, human circadian locomotor output cycles kaput gene; CRC, colorectal carcinoma; shRNA, small hairpin RNA.

In order to investigate the role of hClock in CRC, the effects of upregulation or knockdown of hClock expression on the proliferation of CRC cells were examined. A lentivirus expression system was used to overexpress exogenous hClock in SW480 cells, which were found to have a relatively low endogenous hClock expression compared with that of the loading control; in addition, hClock was confirmed to be significantly upregulated in transfected cells compared with that of the control (P<0.05) (Fig. 1A and B). As shown in Fig. 1C, overexpression of hClock had a significant effect on promoting cellular proliferation in SW480 cells by four days post-transfection (P<0.05). Endogenous hClock expression in CRC SW620 cells was successfully knocked down using lentivirus-mediated hClock shRNAs (Fig. 1D and E). The results showed that knockdown of endogenous hClock expression resulted in the significant inhibition of cell proliferation in SW620 cells compared with that of the shRNA control group (P<0.05) (Fig. 1F).

### hClock inhibits apoptosis of CRC cells

**Figure 2:**
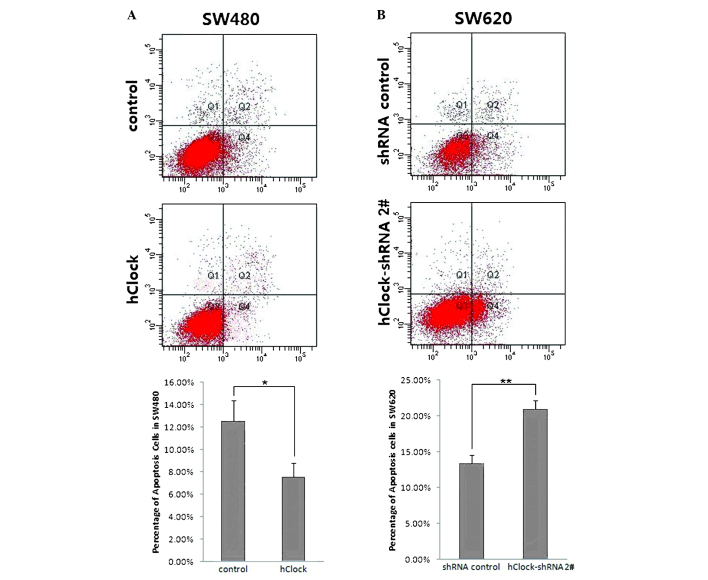
Effects of hClock on cell apoptosis. (A) Flow cytometric analysis was performed to evaluate the impact of hClock on cellular apoptosis. The results showed that overexpression of hClock significantly inhibited SW480 cell apoptosis compared with that in the control group. ^*^P<0.05 vs. control. (B) Flow cytometric analysis showed knock-down of hClock significantly enhanced apoptosis in SW620 cells. ^**^P<0.01 vs. shRNA control. Values are presented as the mean ± standard deviation (n=3). hClock, human circadian locomotor output cycles kaput gene; shRNA, small hairpin RNA.

The effects of hClock upregulation or knockdown on apoptosis were investigated in CRC cells using flow cytometric analysis. The results showed that overexpression of hClock significantly inhibited apoptosis in SW480 cells compared with that of the control group (P<0.05) (Fig. 2A). By contrast, knockdown of hClock expression markedly enhanced apoptosis in SW620 cells compared with that of the shRNA-transfected control group (P<0.01) (Fig. 2B). These results indicated that hClock inhibited CRC cell apoptosis and hClock knockdown may, at least in part, have contributed to increased spontaneous apoptosis in CRC cells *in vitro*.

### Altered expression of hClock is associated with changes in the expression of apoptosis-associated proteins Bax, Bid and p-AKT

**Figure 3:**
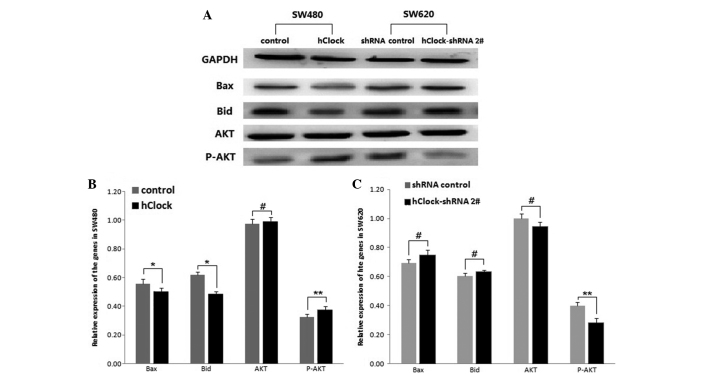
Association between expression of hClock and that of angiogenesis-associated genes in CRC cells. (A) Western blot analysis of Bax, Bid, AKT and p-AKT expression in SW480 cells with hClock overexpression and in SW620 cells with hClock knock-down. (B and C) Densitometric quantification data (expressed as the intensity ratio of the objective genes to GAPDH) calculated from the western blot analysis of proteins in (B) SW480 cells overexpression hClock and (C) SW620 cells with downregulated expression of hClock. Values are presented as the mean ± standard deviation (n=3). ^*^P<0.05, ^**^P<0.01 and #P>0.05 vs. control or shRNA control. hClock, human circadian locomotor output cycles kaput gene; CRC, colorectal carcinoma; shRNA, small hairpin RNA; Bax, B cell lymphoma 2 (Bcl-2)-associated X protein; Bid, Bcl-2 homology 3 interacting domain death agonist; p-AKT, phosphorylated AKT.

In order to further investigate the association between hClock expression and CRC cell apoptosis, the expression of certain apoptosis-associated proteins, including Bax, Bid, AKT and p-AKT, in CRC cells with hClock overexpression or silencing. As shown in Fig. 3A and B, expression levels of Bax and Bid were significantly decreased in SW480 cells overex-pressing hClock compared with those of the vector only-treated SW480 cells (P<0.05), whereas upregulated hClock resulted in a significant increase in p-AKT expression (P<0.01). By contrast, silencing of endogenous hClock expression resulted in a marked decrease in p-AKT expression compared with that of the shRNA-transfected controls (P<0.01) (Fig. 3A and C), whereas Bax and Bid expression levels were not significantly altered. Total AKT expression levels were unaffected by changes in hClock expression (P>0.05) (Fig. 3A–C).

### Overexpression of hClock promotes growth of CRC cells in vivo

**Figure 4:**
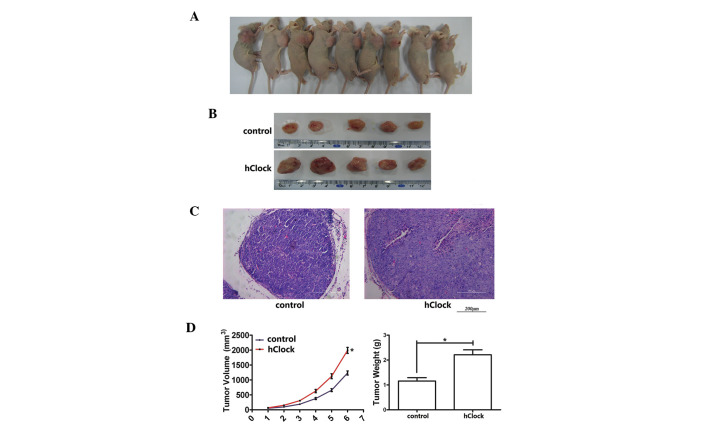
Overexpression of hClock promotes SW480 CRC cell growth *in vivo*. (A) Representative mice that developed tumors following injection of SW480 CRC cells overexpressing hClock. (B) Representative resected tumors derived from subcutaneous transplantation of control SW480 CRC cells or hClock-overexpressing SW480 cells into mice. (C) Representative hematoxylin and eosin staining images of tumor specimens derived from B. (D) Overexpression of hClock enhanced tumor growth. Upregulated expression of hClock accounted for a 61.43% increase in tumor volume and a 91.38% rise in tumor weight at day 42 (^*^P<0.01). Values are presented as the mean ± standard deviation (n=3). ^**^P<0.01 vs. control. hClock, human circadian locomotor output cycles kaput gene; CRC, colorectal carcinoma.

In order to examine whether hClock has an effect on CRC cell growth *in vivo*, a xenograft model of CRC cells in BALB/c nude mice was performed. SW480 CRC tumor cells, which were specifically transduced with hClock or the control vector only, were implanted subcutaneously into the right flank of the nude mice (10 mice/group; 1×10^6^ cells/injection). The volume of the developed tumors was measured once a week for six weeks. All the animals had developed a subcutaneous tumor at one week post-injection of transfected-CRC cells. The mean tumor volume was higher in the hClock over-expression group at two weeks post-injection compared with that of the control group; in addition, the difference in mean tumor volume increased between the two groups from two to six weeks post-transplantation, with significant differences observed at six weeks. Furthermore, upregulated expression of hClock accounted for a 61.43% increase in tumor volume and a 91.38% increase in tumor weight at day 42 post-injection compared with that of the control group (Fig. 4); the developed tumor volume and weight were 1990.0±101.4 mm^3^ and 2.21±0.20 g, respectively, in the hClock overexpression group, compared with 1232.7±69.2 mm^3^ and 1.16±0.14 g in the control group, respectively (P<0.01).

## Discussion

CRC is one of the most prevalent causes of malignancy-associated mortality worldwide. Despite improvements in surveillance and clinical treatment strategies for CRC, the mortality remains high (15). It is therefore important to identify the factors which are associated with CRC tumor progression and the mechanisms by which they proceed (29).

A previous study demonstrated that expression of the circadian gene hClock was significantly increased in CRC patients compared with that in adjacent normal tissue and was strongly correlated with the late stages of TNM classification (18). It was also shown that genetic variants in the hClock gene have a significant effect on the risk of mortality in CRC patients (22). Overall, these studies indicated that hClock overexpression may be a potential biomarker for CRC and therefore, the role of this gene in CRC requires further elucidation.

In the present study, in order to further investigate the role of hClock in promoting CRC progression, hClock was exogenously overexpressed in SW480 CRC cells, which have a relatively low endogenous hClock expression, through transfection of a lentivirus hClock expression system. Overexpression of hClock was found to accelerate SW480 cell proliferation as well as inhibit cell apoptosis. A xenograft model was then used to evaluate the function of hClock in promoting CRC cell growth *in vivo*. The results showed that tumor volume and weight were significantly increased in mice in the hClock overexpression group compared with those injected with control SW480 cells by six weeks post-injection. This therefore suggested that hClock had an active role in promoting CRC proliferation and development *in vitro* and *in vivo*.

It remained to be elucidated whether downregulation of hClock had an effect on CRC cell growth. Therefore, in the present study, an shRNA plasmid targeting hClock was constructed and stably transfected into SW620 CRC cells in order to investigate the effect of hClock silencing on the growth of CRC cells. MTT assays revealed that silencing hClock with shRNA significantly reduced SW620 cell proliferation and enhanced apoptosis *in vitro*. These data provided evidence for the function of hClock as a key regulator of CRC cell growth, which therefore indicated that hClock may be a promising target for CRC treatment.

Circadian genes are important for regulating certain downstream clock-controlled genes (CCGs), including a variety of tumor-associated genes (30). In the present study, in order to further explore the underlying mechanism of the positive role of hClock in CRC progerssion, the expression levels of apoptosis-associated proteins were examined in response to hClock dysregulation. The results showed that hClock overexpression resulted in a significant decrease in Bax and Bid expression as well as a significant increase in p-AKT expression. By contrast, hClock silencing resulted in a significant reduction in p-AKT expression; however, total AKT levels were unaffected by up- or downregulation of hClock. Apoptosis, or programmed cell death, is a normal cellular function which controls excessive proliferation by eliminating ‘unnecessary’ cells; cancer cells are known to develop certain mechanisms in order to avoid apoptosis and prolong their survival (31). Bax and Bid proteins are members of the Bcl-2 family and function as promoters of cell apoptosis (32). Following an appropriate stimulus, the Bax or Bid product primarily enhances apoptotic cell death (33). By contrast, AKT functions in an anti-apoptotic pathway and is activated by a dual regulatory mechanism, which involves translocation to the plasma membrane as well as phosphorylation at Thr308 and Ser473 (34). The mechanism by which activated AKT (p-AKT) protects cells from death is multifactorial. Through activating or inhibiting several downstream components of the cell death machinery, including Bcl-2-associated death promoter, caspase-9, fork-head homologue in rhabdomyosarcoma, nuclear factor-κB and p53, via phosphorylation, p-AKT regulates numerous cell activities, including cell proliferation, differentiation, apoptosis and migration (31). The results of the present study indicated that hClock promoted the activity of anti-apoptosis gene p-AKT and inhibited the expression of the pro-apoptotic genes Bax and Bid, thereby inhibiting the apoptosis of CRC cells. By contrast, targeted inhibition of hClock expression partially reduced the levels of p-AKT, which may be used as a novel target of CRC therapy. Overall, these data suggested that the p-AKT, Bax and Bid may work as CCGs and have important roles in regulating cell apoptosis under the control of the circadian gene hClock.

In conclusion, the results of the present study demonstrated that overexpression of the circadian gene hClock had an enhancing role in CRC progression and may inhibit apoptosis in CRC cells *in vitro* and *in vivo*. In addition, silencing hClock in CRC cells had an opposite effect. These results indicated that hClock was functionally important in regulating the progression of CRC and may serve as a novel target for CRC therapy.
